# Center volume and the outcomes of percutaneous transluminal angioplasty and stenting in patients with symptomatic intracranial vertebrobasilar stenoses: A meta-analysis

**DOI:** 10.1371/journal.pone.0200188

**Published:** 2018-07-10

**Authors:** Ying Mao, Guangxian Nan

**Affiliations:** Department of Neurology, China-Japan Union Hospital of Jilin University, Changchun, China; Universitatsklinikum Freiburg, GERMANY

## Abstract

**Background:**

Evidence for the preventative effects of percutaneous transluminal angioplasty and stenting (PTAS) on the recurrence of stroke in patients with severe intracranial vertebrobasilar stenoses (IVBS) varies, and the influence of study characteristics on the study outcomes have not been determined.

**Methods:**

A study level based meta-analysis was performed to investigate the influence of baseline characteristics on the 30-day and follow-up stroke recurrence or death in symptomatic IVBS patients receiving PTAS. Relevant single center studies were retrieved by searching PubMed and Embase. A random effect model was applied to synthesize the outcomes. Meta-regression and subgroup analyses were performed to evaluate the potential influence of study characteristics on outcomes.

**Results:**

Fifteen cohort studies comprising 554 symptomatic IVBS patients were included. PTAS was associated with an 8% incidence of stroke recurrence or death (95% CI: 5% to 12%) in IVBS patients within 30 days, and 8 per 100 person-years (95% CI: 5 to 11 per 100 person-years) of cumulative stroke recurrence or death during follow-up. Meta-regression indicated that the center volume, as defined by the numbers of cases per year, was negatively correlated with 30-day (regression coefficient = -0.09, p = 0.02) and follow-up (regression coefficient = -0.60, p = 0.01) stroke recurrence or death. Age, gender, or comorbidities have no significant effect on the outcomes.

**Conclusions:**

Centers of higher procedural volume may be associated with better clinical outcomes for symptomatic IVBS patients receiving PTAS.

## Introduction

Although ischemic events in the anterior cerebral circulation contribute to about 80% of all cerebral ischemic events, patients with impairment posterior cerebral circulation were considered to be at higher risk of recurrence of cerebral ischemic events [1]. Stroke was reported to recur in 8–10% of patients with a previous minor stroke or transient ischemic attach (TIA) [2]. However, an annual recurrence of 15% was reported in patients with impaired posterior cerebral circulation, and particularly those with symptomatic intracranial vertebrobasilar stenoses (IVBS), despite administration of the optimal medical therapy [3, 4]. Indeed, in a previous large study of Warfarin-Aspirin Symptomatic Intracranial Disease (WASID), stroke recurred in the posterior circulation in 23% and 10% of patients assigned to aspirin and warfarin therapy, while stroke in the anterior circulation recurred in 8% and 2% of these groups, respectively [5]. Novel strategies capable of preventing stroke are urgently sought, particularly for patients with symptomatic IVBS [6].

Early case reports and series indicated that endovascular treatment with percutaneous transluminal angioplasty and stenting (PTAS) may effectively prevent stroke in patients with severe intracranial arterial stenoses [7]. However, subsequent randomized controlled trials (RCTs) reported that PTAS was associated with poorer clinical outcomes in patients with severe intracranial arterial stenosis (ICAS) when compared with the aggressive medical therapy [8]. Indeed, the Stenting and Aggressive Medical Management for Preventing Recurrent Stroke in Intracranial Stenosis (SAMMRIS) trial, in which 35% of enrolled patients had IVBS, was stopped prematurely because the 30-day rate of stroke or death was significantly higher in the PTAS group with the Wingspan stent system as compared with the medical group [9]. Moreover, a recent RCTs aiming to compare the efficacy of PTAS and medical treatment in patients with vertebral artery stenoses was also halted early because stenting of symptomatic vertebral artery stenosis was associated with a major periprocedural vascular complication [10]. However, the limited numbers of available RCTs makes it difficult to determine the key determinants of the poor clinical outcomes in IVBS patients undergoing PTAS. Moreover, the efficacies of PTAS in patients with IVBS varied considerably between previous cohort studies [11–27]. Moreover, the factors that contribute to clinical outcomes in patients with IVBS that receive PTAS have not been determined. In this study level based meta-analysis, we aimed to investigate the influence of center and baseline patient characteristics on 30-day and follow-up outcomes in symptomatic IVBS patients receiving PTAS, focusing on the volume of the individual center included.

## Methods

The Meta-Analysis of Observational Studies in Epidemiology (MOOSE) [28] protocol and Cochrane Handbook [29] guidelines were followed throughout the design, implementation, analysis, and reporting of this systematic review and meta-analysis.

### Literature search

We systematically searched the electronic databases PubMed (https://www.ncbi.nlm.nih.gov/pubmed/) and Embase (Ovid from Wolters Kluwer) for relevant records using the search strategies outlined in **S1 File**. We used this broad strategy in order to incorporate as many relevant studies as possible. The search was limited to studies clinical studies published in English. The reference lists of original and review articles were also manually screened to identify additional records. The final literature search was performed on April 20th, 2018.

### Inclusion and exclusion criteria

Studies were included in our systematic review and meta-analysis if they met the following criteria: 1) published as full-length article; 2) reported as a single center cohort study in humans (prospective or retrospective design); 3) including at least ten consecutive cases with symptomatic IVBS undergoing PTAS; 4) with a follow-up duration of ≥ 1 month; 5) reporting the composite outcome of recurrence of ischemic stroke or all-cause death within 30 days of the PTAS procedure or during subsequent follow-up. Studies published as editorial, letters, or conference abstracts were excluded. Studies including patients without IVBS, with specific requirements for stents used and subsequently included non-consecutive cases of IVBS were excluded, in addition to studies including IVBS patients that were confirmed to be caused by dissections, or including patient that underwent surgical treatment such as bypass.

### Data extraction and quality evaluation

The literature search, data extraction, and quality assessment were independently performed by the two authors according to the predefined inclusion criteria. Discrepancies were resolved by consensus and discussion. To define the potential influence of center volume on the PTAS outcome, we extracted each consecutive IVBS patient’s data and the duration of the recruitment and procedures. The center volume was defined as the number of consecutive IVBS patients that received PTAS divided by the time of the recruitment and procedures (cases per year). Therefore, the extracted data included: 1) author, publishing year, design, and located country of each study; 2) baseline characteristics of the included patients: numbers, mean ages, proportions of male, hypertensive, diabetic, or hyperlipidemic, proportions of current smokers, proportions with lesions of basilar arteries; 3) other study characteristics, such as center volume as defined above, stent systems used and follow-up durations; 4) outcome data, including the patients with recurrent ischemic stroke or all-cause death within 30 days after the PTAS procedure or during subsequent follow-up. The quality of the included studies was evaluated using the modified Newcastle-Ottawa Scale, which addresses the following aspects [30], including the manner of data collection (prospective or retrospective), methods of detecting vascular stenoses, definitions of stroke recurrence and deaths, follow-up methods, and loss to follow-up. Each aspect was assigned 1 point, with 5 points as the highest score.

### Statistical analysis

The purpose of the study was to evaluate the potential effects of center volume on the composite outcomes of stroke recurrence and death within 30 days after the PTAS procedure or during the follow-up. Therefore, the numbers of patients with the above outcomes were calculated. If the same patients suffered from both stroke recurrence and subsequent death, they were counted only once. The incidence of stroke recurrence and death within 30 days was calculated as follows: the number of patients with events within 30 days divided by the total numbers of patients included in each study (events / total patients). The incidence of stroke recurrence and death during follow-up was calculated as follows: number of patients with events at the end of the follow-up divided by total person-years (events / person-years). These outcomes were synthesized with a random-effect model to incorporate the potential heterogeneities among the included studies [29]. The weight (%) of each study was calculated based on the incidence of events and sample size in each study via the DerSimonian-Laird methods as previously described [31]. We used the Cochrane’s Q test to formally assess the inter-study heterogeneity, and a p value < 0.10 indicated the potential existence of significant heterogeneity [32, 33]. The I^2^ statistic was also calculated to assess the contribution of heterogeneity, as opposed to chance, to total variation [34]. A value of I2 > 50% indicated significant heterogeneity. Meta-regression and predefined subgroup analysis were performed to identify the source of heterogeneity, particularly focusing on the potential influence of center volume. Median and interquartile values of continuous variables were used as cut-off values for grouping studies. Potential publication bias was assessed by the visual inspection of the symmetry of the funnel plots, and the results of the Egger regression asymmetry test [35]. We used STATA software (Version 12.0; Stata Corporation, College Station, TX) for these statistical analyses.

## Results

### Database searching

**Fig 1:**
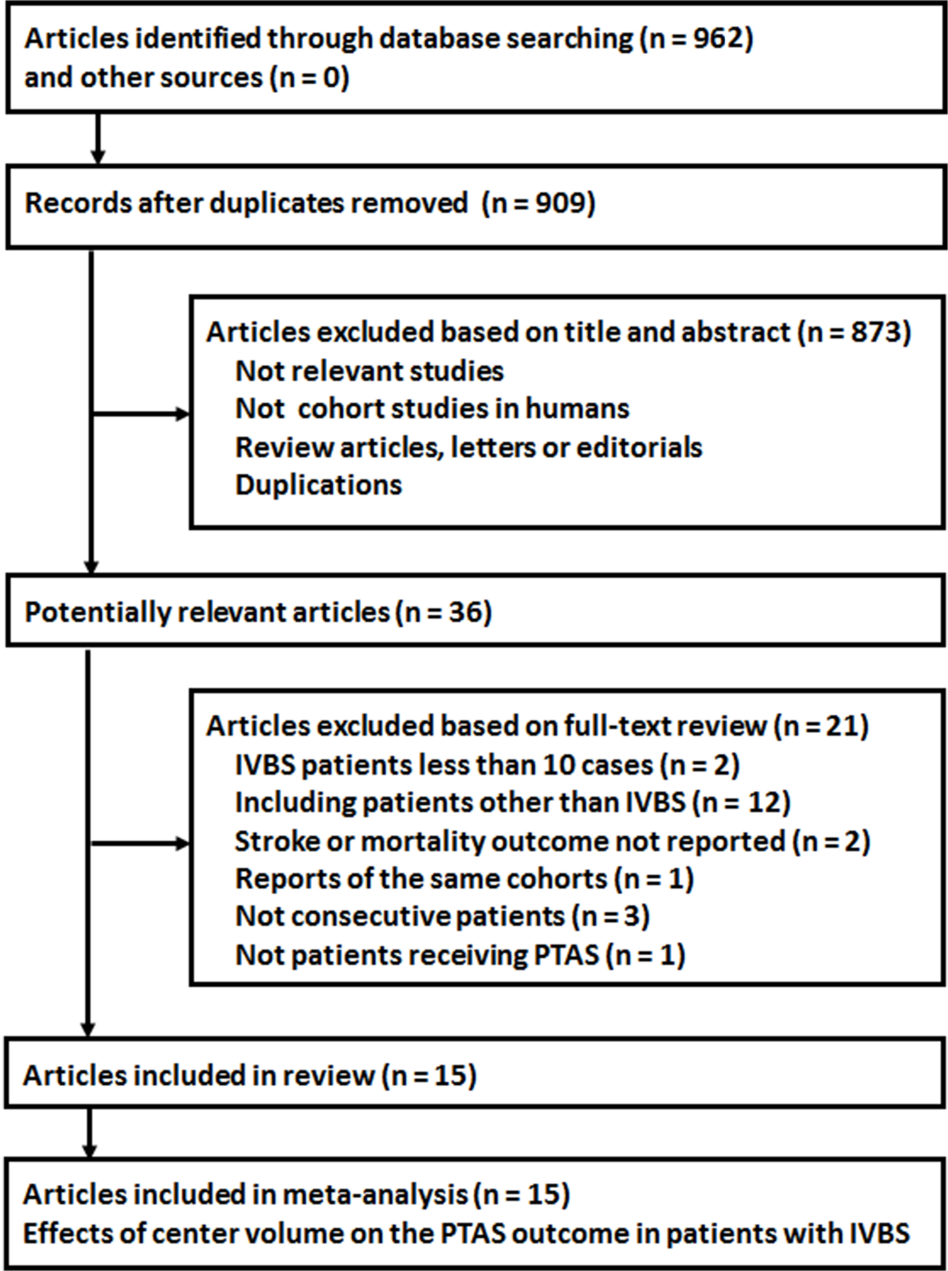
The flowchart of database searching.

Relevant literature was identified as described in **Fig 1**. Briefly, 962 records were retrieved from the initial search of PubMed and Embase databases. After removing duplicate reports and primary screening of the included articles’ titles and abstracts 36 records were considered to be relevant. After full-text review, 15 studies [11–22, 24, 25, 27] were finally included in the current systematic review and meta-analysis after further exclusion of 21 records that patients without IVBS, enrolled non consecutively, included patients not receiving PTAS, did not report relevant outcomes, or enrolled duplicate cohorts.

### Study characteristics

The 15 included cohort studies [11–22, 24, 25, 27] enrolled a total of 554 symptomatic IVBS patients that underwent PTAS procedures. The characteristics of the included studies are listed in **Table 1**. These studies were published between the year 2000 and 2016. Most were retrospective cohort studies, except four [19, 21, 25, 27] which were prospective cohort studies. The study centers were distributed in North America [11, 12, 15–17], Europe [14, 19, 20], Asia [13, 21, 24, 25, 27], Argentina [22] and Australia [18]. The mean age of the included patients ranged from 59.0 to 68.3 years, and patient groups were 58.8% to 100% male. The mean stenoses of the targeted lesions ranged from 67.0% to 92.3%, and the proportions of patients with basilar stenoses ranged from 32% to 100%. The follow-up duration ranged from 1 to 43.5 months. Center volume differed significantly between studies, ranging from 2 to 97 patients per year. The early studies applied coronary stents [11–16, 18] for the PTAS procedures in patients with symptomatic IVBS, while the Wingspan and Apollo stent systems were applied in more recent studies [21, 24, 25, 27]. The quality of the included studies was generally optimal, with quality scores ranging from 4 to 5 points.

**Table 1:** Baseline characteristics of included studies.

Study	Design	Location	Patient Number	Mean age	Male	HTN	DM	HD	Smoking	Mean stenosis	Center volume	Basilar artery lesions	Stents system	Follow-up	Quality scores
				years	%	%	%	%	%	%	case/year	%	%	months	
Gomez 2000	RC	USA	12	62.6	83.3	NR	NR	NR	NR	71.4	12	100	Coronary stents	5.9	4
Levy 2001	RC	USA	11	62.9	100	72.7	9	27.3	45.5	> 70	5.5	72.7	Coronary stents	4	4
Kim 2005	RC	Korea	17	64	58.8	100	35.3	29.4	35.3	76.1	5.7	46.2	Coronary stents	17	4
Weber 2005	RC	Germany	21	67	71.4	NR	NR	NR	NR	92.3	11	40.9	Coronary stents	9	4
Yu 2005	RC	USA	18	69	83.3	83	50	72	33	79.6	4.5	100	Coronary stents	12.1	4
Abruzzo 2007	RC	USA	10	68.3	80	90	40	30	20	80.7	2.5	100	Coronary stents	31	4
Steinfort 2007	RC	Australia	13	60	100	NR	NR	NR	NR	67	6.5	38.5	Paclitaxel-coated stents	10.3	4
Fiorella 2007	RC	USA	44	64.8	79.5	NR	NR	NR	NR	82.5	7.3	NR	Velocity, Duet, Tetra and Vision stents	43.5	4
Ralea 2008	PC	France	12	62.6	66.7	91.7	25	100	25	80	2	50	Tsunami, INX, Cérébrence and Wingspan stents	15	4
Seifert 2009	RC	Austria	17	65.9	70.6	NR	NR	NR	NR	NR	3.4	NR	Coronary stents or self-expanding nitinol stents	12.7	4
Povedano 2010	RC	Argentina	25	63	84	84	24	60	40	NR	2.5	32	AVE INX and AVE GFX; Cypher, Sonic, and Velocity; Express and Neuroform; Taxus Express; Herculink and MultiLink Pixel; and Wingspan stents	12	4
Jiang 2010	PC	China	139	59	92.1	78.4	28.8	80.6	66.2	81	19.8	50	Wingspan stents	24.9	5
Li 2012	RC	China	30	60.3	83.3	86.7	40	16.7	46.7	82.3	10	46.7	Wingspan stents	17.8	4
Wang 2015	PC	China	88	62.6	75	77.3	52.3	50	34.1	82.2	14.6	40.9	Wingspan stents	29.3	5
Liu 2016	PC	China	97	58.5	82.1	82.4	28.9	44.3	61.9	84.2	48.5	53.6	Apollo and Wingspan stents	1	5

RC, retrospective cohort; PC, prospective cohort; HTN, hypertension; DM, diabetes mellitus; HD, hyperlipidemia; NR, not reported

### Stroke recurrence or death within 30 days or during follow-up after PTAS

**Fig 2:**
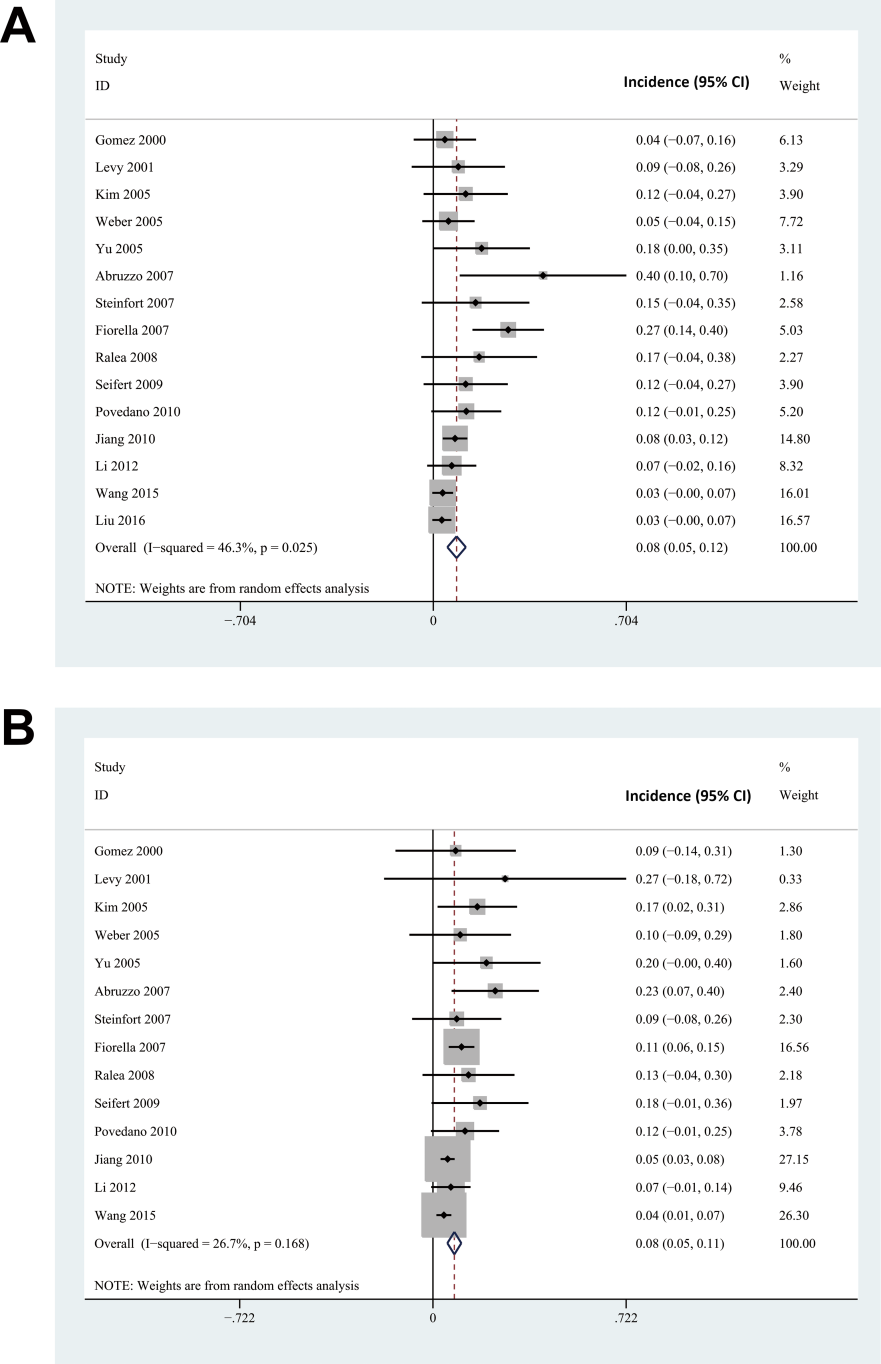
The forest plots of the meta-analysis of incidences of combined outcome of stroke recurrence and death in symptomatic IVBS patients after PTAS. A, forest plot for the incidence of combined outcome of stroke recurrence and death within 30 days after PTAS; forest plot for the incidence of combined outcome of stroke recurrence and death during follow-up (per person year).

By combing the results of 15 included cohort studies with a random effect model, the meta-analysis indicated PTAS procedure was associated with an 8% incidence of stroke recurrence or death (95% CI: 5% to 12%) in IVBS patients within 30 days after the procedure. Considerable heterogeneities in incidence were noticed (Cochrane’s Q test: p = 0.03, I^2^ = 46.3%), varying from 3% to 40% among included studies (**Fig 2A**). Stroke recurrence or death during follow-up was reported in 14 studies [11–22, 24, 25]. The pooled results indicated that the risk of cumulative stroke recurrence or death during follow-up was 8 per 100 person-years for IVBS patients receiving PTAS (95% CI: 5 to 11 per 100 person-years). Although only moderate heterogeneity was noticed among the included studies (Cochrane’s Q test: p = 0.17, I^2^ = 26.7%), the risk of stroke recurrence or death during follow-up varied considerably from 4 to 23 per 100 person-years (**Fig 2B**).

### Influence of study characteristics on stroke recurrence or death after PTAS

**Fig 3:**
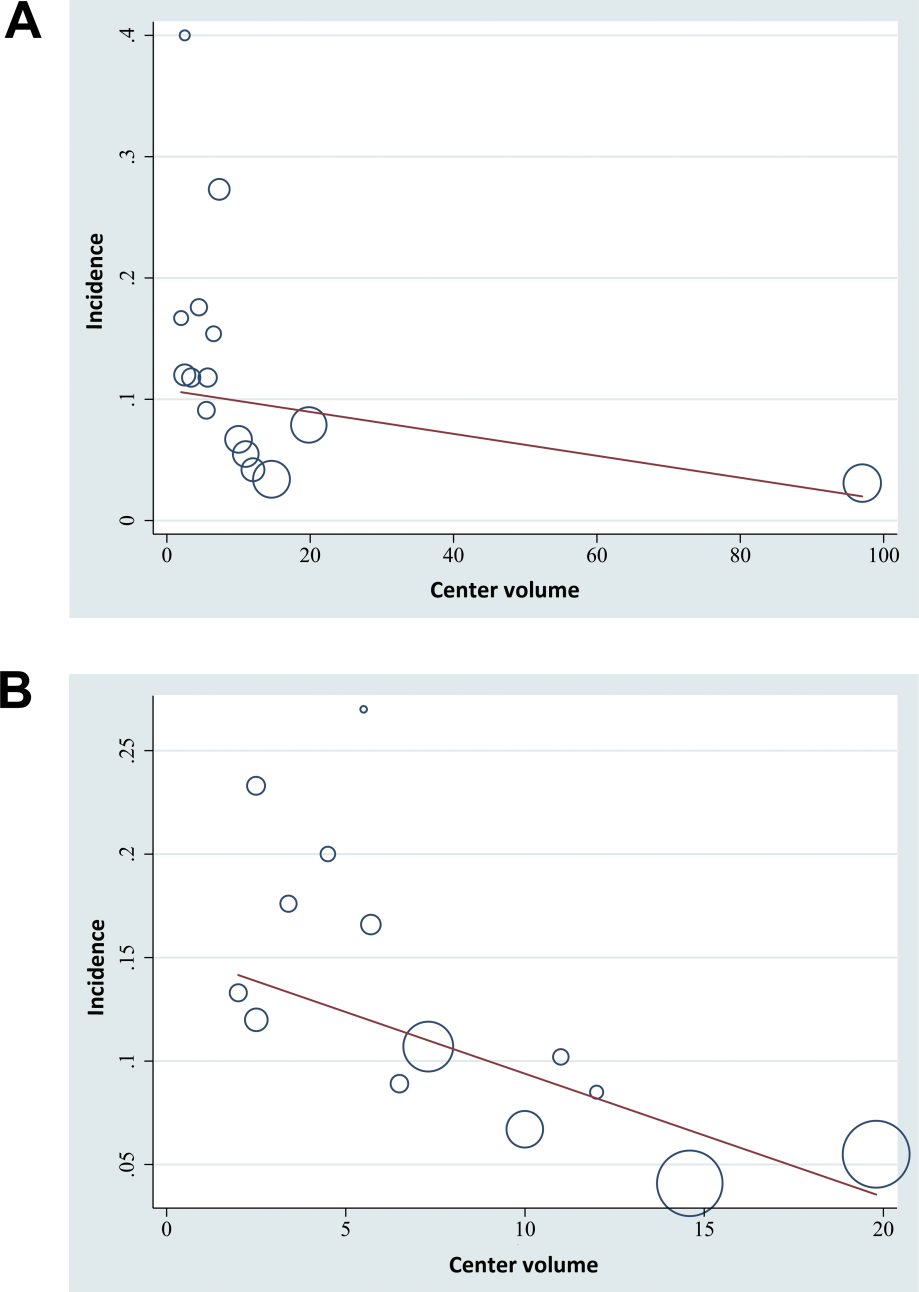
Influence of center volume on the risks of stroke recurrence or mortality in symptomatic IVBS patients after PTAS. A, correlation of center volume with the incidence of stroke recurrence and death within 30 days after PTAS; B, correlation of center volume with the incidence of stroke recurrence and death during follow-up (per person year).

We subsequently evaluated the potential influence of study characteristics on the rate of stroke recurrence or death after PTAS via meta-regression analyses. Results showed that the center volume of the included studies as defined by the numbers of cases per year was negatively correlated with both the risk of stroke recurrence or death within 30 days after PTAS (regression coefficient = -0.09, 95% CI: -0.16 to -0.02, p = 0.02; **Table 2 and Fig 3A**) and that during follow-up (regression coefficient = -0.60, 95% CI: -1.06 to -0.13, p = 0.01; **Table 2 and Fig 3B**). Other study characteristics, such as the mean age of enrolled patients, the proportion of male hypertensive, diabetic, hyperlipidemic, or smoker patients, or the proportion of patients with the basilar artery lesions were not significantly associated with outcomes.

**Table 2:** Impact of study characteristics on the outcomes stroke recurrence or death within 30 days after PTAS or during clinical follow-up.

	30 days after PTAS (%)			During clinical follow-up (per100 person-year)		
	Coefficient	95% CI	p	Coefficient	95% CI	p
**Mean age (years)**	1.28	-0.32 to 2.89	0.10	1.59	-0.42 to 3.60	0.11
**Males (%)**	0.02	-0.49 to 0.52	0.93	-0.16	-0.73 to 0.42	0.55
**HTN (%)**	0.39	-0.33 to 1.11	0.25	0.61	-0.12 to 1.35	0.09
**DM (%)**	-0.08	-0.45 to 029	0.64	-0.14	-0.66 to 0.38	0.55
**HD (%)**	0.05	-0.14 to 0.24	0.55	-0.04	-0.26 to 0.19	0.72
**Center volume (case per year)**	-0.09	-0.16 to -0.02	0.02	-0.60	-1.06 to -0.13	0.01
**Basilar artery lesions (%)**	0.21	-0.08 to 0.50	0.14	0.10	-0.06 to 0.26	0.13

HTN, hypertension; DM, diabetes mellitus; HD, hyperlipidemia

### Impact of center volume on the risks of stroke recurrence or death after PTAS

The influence of center volume on the risks of stroke recurrence or death after PTAS in symptomatic IVBS patients was further confirmed by subgroup analyses. The first quartile, the median and the third quartile (4, 7, and 10 cases per year) were used as cut-offs for grouping of the patients. Studies with center volumes of ≥ 4 cases per year reported significantly lower risk of stroke recurrence or death during the follow-up (p for subgroup difference = 0.02), but not for that within 30 days after PTAS (p for subgroup difference = 0.10, Table 3). Studies with center volumes of ≥ 7 and 10 cases per year reported significantly lower risks of stroke recurrence or death within 30 days after PTAS (p for subgroup difference both < 0.05) or during follow-up (p for subgroup difference both < 0.01, **Table 3**).

**Table 3:** Impact of center volume on the clinical outcomes of PTAS.

Case volume (case per year)	30 days after PTAS				During clinical follow-up			
	Studies (patients), n	I^2^	Incidence (%)	p for subgroup	Studies (patients), n	I^2^	Incidence (per 100 peson-year)	p for subgroup
**First Quartile**								
< 4	4 (64)	0%	15 (6–23)		4 (64)	0%	16 (8–24)	
≥ 4	11 (490)	46%	7 (3–11)	0.10	10 (393)	39%	7 (4–9)	0.02
**Median**								
< 7	8 (123)	0%	14 (8–20)		8 (123)	0%	16 (10–22)	
≥ 7	7 (431)	60%	6 (3–10)	0.04	6 (334)	14%	6 (4–8)	<0.01
**Third Quartile**								
< 10	10 (188)	16%	14 (9–19)		10 (188)	0%	13 (9–16)	
≥ 10	5 (366)	0%	4 (2–7)		4 (269)	0%	5 (3–7)	<0.01

### Publication bias

Visual inspection indicated symmetrical funnel plots, implying a low chance of significant publication biases. These results were further confirmed by Egger’s regression tests (p = 0.52 for the outcome within 30 days, and p = 0.71 for the outcome during follow-up).

## Discussion

In this meta-analysis of all available cohort studies, we evaluated the potential determinants of 30-day and cumulative stroke recurrence or death in patients with symptomatic IVBS that received PTAS procedures. We found that the volume of each center, as indicated by the numbers of cases enrolled per year, was negatively correlated to the stroke recurrence or death within 30 days of PTAS and during the subsequent follow-up. These results were further confirmed by subgroup analyses, grouping by stroke volume quartiles, which indicated that studies with larger volumes reported better clinical outcomes in IVBS patients receiving PTAS. Other characteristics, including the mean ages of the patients, the proportion of male, hypertensive, diabetic, hyperlipidemic, or currently smoking participants, or the proportion with basilar artery lesions were not significantly associated with outcomes. These results demonstrate that PTAS procedures performed in high volume centers may have better preventative efficacy for stroke recurrence or death in patients with IVBS. This observation suggests that the experience of the operator may be a key determinant of the clinical efficacy of PTAS procedure.

Since disappointing results were reported by RCTs, suggesting that PTAS may be associated with more frequent periprocedural and follow-up stroke recurrence and mortality [8–10], efforts have been made to characterize outcome determinants. In a previous retrospective cohort study of 583 patients with symptomatic ICAS undergoing PTAS, age, comorbidities of diabetes, and basilar arty lesions were potential independent contributors to increased risk of serious in-hospital adverse events [26]. However, our study level based meta-regression analyses did not find the above factors to be significant associated with clinical outcomes in patients receiving PTAS. Despite differences in the populations evaluated, we could not exclude potential determinative effects of these above factors, and the influence of these factors should be further investigated in cohort studies or meta-analyses of adequate data from individual patients. Future studies are warranted. Interestingly, previous studies also showed that the experience of the operator may also be a key determinant of the PTAS outcome. In a prospective cohort study of 95 patients with ICAS, a learning curve for mastering the safety precautions of Wingspan stenting for ICAS was identified, reflecting improved development of the techniques in each surgeon with each surgery performed [36]. Also, a retrospective study of 188 patients identified operator experience to be an independent predictor of periprocedural complications in patients with ICAS receiving PTAS [37]. Our analyses confirmed and extended these findings. To the best of our knowledge, our study is the first meta-analysis to systematically evaluate the potential influence of study characteristics on the clinical efficacy of PTAS in patients with IVBS. More importantly, we identified a potential negative association between center volume and the risk of 30-day and cumulative stroke recurrence and death during follow-up, highlighting that PTAS performed by an experienced operator in a qualified center may represent the most effective treatment for patients with symptomatic IVBS. As indicated by the results of a recently published subgroup analysis of SAMMPRIS [38], for patients with IVBS, the incidence of the combined outcome of recurrent stroke and death was reported to be 4.9 per 100 person-years in patients allocated to the aggressive medical therapy, while it was 13.5 per 100 person-years in PTAS group. Interestingly, in the two studies with the largest center volume [21, 25], the incidence of the combined outcome of recurrent stroke and death were reported to be 4 and 5 per 100 person-years after PTAS, respectively. These data, together with our finding that centers with higher procedural volume may be associated with better clinical outcomes for symptomatic IVBS patients receiving PTAS, may indicate that PTAS for IVBS may achieve equal or even better clinical efficacy than aggressive medical therapy where performed by experienced physicians at centers with large procedural volumes. Moreover, due to a lack of recourses and compliance, the goal of aggressive medical therapy and lifestyle intervention as applied in RCTs could not be achieved in real-world clinical practice. Therefore, if performed by experienced operators at centers with large procedural volumes, PTAS may confer equal or even better clinical efficacy than aggressive medical therapy in the real world. Further studies are needed to confirm these results.

Despite these strengths, our conclusions are limited by the scope of this study. Firstly, only few cohort studies were available for this current meta-analysis and meta-regression study, which provide inadequate statistical power to detect the significant influence of all relevant study characteristics to the efficacy of PTAS in IVBS patients. The influence of characteristics such as the age, gender, and comorbidities on the clinical efficacy of PTAS should be optimally evaluated in a large cohort study or meta-analysis of individual patients. Secondly, due to the limited number of cohort studies included, we were not able to confirm whether the influence of center volume on clinical outcomes of PTAS in symptomatic IVBS patients was independent from other potential factors. If adequate numbers of cohort studies can be identified in the future, multivariate analyses will be required. Thirdly, differences such as location of the lesions, severity of stenoses, and stent systems applied may contribute to the heterogeneity among the included studies, which may further confound the results. Indeed, the IVBS intervention location may affect the outcomes stroke recurrence and death. However, the influence of intervention location on the stroke recurrence and death could not be evaluated on the study-level because IVBS intervention location was rarely reported. Finally, our study is hypothesis generating. The determination of the standards for the qualification of the operators and centers of PTAS for symptomatic IVBS patients should be intensively discussed and investigated in real world practice to improve the comprehensive care of these patients.

## Conclusions

In conclusion, our analyses indicated that PTAS procedures performed in high volume centers may have better preventative efficacy for stroke recurrence or death in patients with IVBS. These results highlight that the experience of the operator may be a key determinant of the clinical efficacy of PTAS procedure. Future studies are needed to determine the comparative efficacy of PTAS and aggressive medical therapy in certain subgroup of patients with ICAS with PTAS performed by experienced operators in centers with large procedural volumes. Moreover, in addition to RCTs, the comparative efficacy of PTAS performed by experienced operators and aggressive medical therapy should be investigated in real world studies.

## Supporting information

S1 File Search strategies. (DOCX)

S2 File PRISMA checklist. (DOC)
