# Supplementary material for: Center volume and the outcomes of percutaneous transluminal angioplasty and stenting in patients with symptomatic intracranial vertebrobasilar stenoses: A meta-analysis
Source: PLoS One. 2018 Jul 10;13(7):e0200188. doi: 10.1371/journal.pone.0200188 (PMC6039023; doi:10.1371/journal.pone.0200188)
Supplement: S1 File — (DOCX) [file pone.0200188.s001.docx]

**Appendix 1. PubMed search strategy**

| Search | Query | Papers |
| --- | --- | --- |
| #1 | "Angioplasty"[Mesh] | [58876](https://www.ncbi.nlm.nih.gov/pubmed/?cmd=HistorySearch&querykey=11) |
| #2 | (((Transluminal[tw]) OR Percutaneous[tw]) OR Endoluminal[tw]) | [136852](https://www.ncbi.nlm.nih.gov/pubmed/?cmd=HistorySearch&querykey=12) |
| #3 | ((("cerebral" [tw] AND "revasculariz*" [tw]) OR ("blood vessel"[tw] AND "prosthesis"[tw] AND "implant*"[tw]))) | [433](https://www.ncbi.nlm.nih.gov/pubmed/?cmd=HistorySearch&querykey=13) |
| #4 | #1 OR (#2 AND #3) | [58890](https://www.ncbi.nlm.nih.gov/pubmed/?cmd=HistorySearch&querykey=15) |
| #5 | (stents[tw] OR stent[tw] OR stenting[tw] OR dilatation[tw] OR catheterization[tw]) | [300414](https://www.ncbi.nlm.nih.gov/pubmed/?cmd=HistorySearch&querykey=16) |
| #6 | #4 AND #5 | [28990](https://www.ncbi.nlm.nih.gov/pubmed/?cmd=HistorySearch&querykey=17) |
| #7 | (((intracranial[tw] AND vertebrobasilar[tw] AND (stenoses[tw] OR stenosis[tw]))) OR "Vertebrobasilar Insufficiency"[Mesh]) OR "intracranial arterial disease"[tw] OR "arterial occlusive disease"[tw] | [8044](https://www.ncbi.nlm.nih.gov/pubmed/?cmd=HistorySearch&querykey=18) |
| #8 | #6 AND #7 | [538](https://www.ncbi.nlm.nih.gov/pubmed/?cmd=HistorySearch&querykey=19) |

**Appendix 2. EMBASE (Ovid) search strategy**

| No. | Query | Results |
| --- | --- | --- |
| #9 | #4 AND #7 AND [humans]/lim AND [english]/lim AND [clinical study]/lim AND [2000-2017]/py AND [priority journals]/lim | **424** |
| #8 | #4 AND #7 | **1759** |
| #7 | #5 OR #6 | **10958** |
| #6 | 'vertebrobasilar insufficiency'/exp OR 'cerebral artery disease'/exp | **6968** |
| #5 | 'brain artery'/exp AND (stenosis OR stenoses) | **4243** |
| #4 | #1 OR #2 OR #3 | **581367** |
| #3 | angioplast$ OR pta OR stent$ OR dilatation OR catheter$ | **577433** |
| #2 | 'stent'/exp OR 'balloon dilatation'/exp OR 'balloon catheter'/exp | **174449** |
| #1 | 'angioplasty'/exp | **81363** |
